# Fatal traumatic injury patterns in free-roaming cats: an anatomopathological study of 40 cases

**DOI:** 10.1186/s12917-026-05733-z

**Published:** 2026-07-20

**Authors:** Rubia Avlade Guedes Sampaio, Rodrigo Gonçalves Ferreira, Ícaro Matheus Martins Leite, Isabelle Vieira de Sousa, Gedean Galdino da Cruz Silva, Markyson Tavares Linhares, Wellida Karinne Lacerda, Lucas Rannier Ribeiro Antonino Carvalho, Ricardo Barbosa Lucena

**Affiliations:** 1 Graduate Program in Animal Health and Science, Center for Health and Rural Technology, Universidade Federal de Campina Grande, Patos, Paraíba 58700-000 Brazil; 2 Veterinary Medicine Program, UNIFACISA, Campina Grande, Paraíba, 58411-020 Brazil; 3 PatoBiology Laboratory, Universidade Federal da Paraiba, Areia, PB 58397- 000 Brazil; 4 Department of Physiology and Pharmacology, Karolinska Institutet, Biomedicum, 5B, Karolinska Institutet, Biomedicum, 5B, Solnavägen 9, Stockholm, S-171 77 Sweden

**Keywords:** Bite injury, Feline, Forensic pathology, Hemoperitoneum, Hemothorax, Projectile trauma, Poadkill

## Abstract

**Background:**

Trauma is an important cause of death in domestic cats, particularly in animals with unrestricted outdoor access. However, necropsy-based studies focusing specifically on immediately fatal trauma in free-roaming cats remain limited. This study aimed to characterize fatal traumatic injuries in free-roaming cats through a retrospective anatomopathological review of feline necropsies performed over a 10-year period at the Veterinary Pathology Laboratory of the Universidade Federal da Paraíba, Brazil, based on gross findings, histopathology, and complementary imaging when available.

**Results:**

Among 289 feline necropsies examined between September 2014 and September 2024, 40 cases (13.84%) were classified as immediately fatal traumatic injuries in free-roaming cats. Blunt force trauma predominated (32/40), followed by sharp-force trauma (7/40) and projectile trauma (1/40). Road traffic accident was the most frequent recorded or presumed traumatic mechanism. Lesions most frequently involved the thorax (31/40), and multiple body regions were affected in 25/40 cases. The most common gross findings were extrapulmonary hemorrhage, pulmonary hemorrhage, atelectasis or pulmonary collapse, fracture, hemothorax, hemoperitoneum, visceral herniation into the thoracic cavity, and diaphragmatic rupture. Compared with sharp-force trauma, blunt force trauma was associated with significantly higher EGTN values and with a greater frequency of extrapulmonary hemorrhage, fracture, pulmonary hemorrhage, and abdominal/visceral involvement. Among vehicle-identified road traffic trauma cases, car-related trauma was associated with higher EGTN values and greater multiregional involvement than motorcycle-related trauma. Concurrent non-traumatic lesions were recorded in five cases, and potentially predisposing conditions, most commonly chronic debilitating or neoplastic diseases, were identified in nine.

**Conclusions:**

Immediately fatal trauma in free-roaming cats was dominated by blunt force trauma, most often associated with road traffic accidents, and characterized by extensive thoracic involvement, frequent multiregional distribution, and severe internal damage. Systematic necropsy, supported by histopathology and complementary imaging when available, provided valuable information on lethal lesion patterns, probable traumatic mechanisms, and concurrent non-traumatic conditions. These findings highlight the value of veterinary pathology in the characterization of fatal feline trauma and in the broader contexts of forensic interpretation, animal welfare assessment, and prevention-oriented understanding of trauma in free-roaming cat populations.

## Background

Trauma is a major cause of morbidity and death in companion animals and remains one of the most important categories of fatal injury in domestic cats [1]. In feline patients, traumatic events are associated with a broad spectrum of gross and microscopic lesions, ranging from localized soft tissue injury to severe multisystem damage and death [2]. The type and severity of traumatic injury depend on several variables, including the nature of the external force, the amount of kinetic energy involved, the body region affected, and the manner in which that energy is transmitted through tissues [1, 3, 4]. Among small animals, cats are especially relevant in trauma studies because of their frequent exposure to outdoor hazards and their distinctive patterns of injury and survival after high-impact events [5–9].

In Brazil, free-roaming cats, including semi-domiciled cats with unrestricted outdoor access, stray or rescued cats recovered from the street, and cats with unknown previous ownership history but clear evidence of unrestricted exposure to the outdoor environment, are commonly encountered in diverse settings across the country [10]. In these animals, the risk of traumatic injury is amplified by unrestricted exposure to road traffic, aggression by other animals, falls, crushing injuries, and intentional violence. Previous clinical and epidemiologic studies have shown that blunt force trauma is the predominant mechanism in feline trauma populations, with road traffic accidents representing one of the most important causes of severe injury and death. In contrast, bite-associated sharp-force injuries and projectile trauma are less frequent, but they are often associated with distinctive lesion patterns and, in some cases, substantial forensic relevance [4–12]. In particular, bite-related injuries may produce deep puncture wounds, tearing of soft tissues, muscular disruption, pneumothorax, and fatal hemorrhage, whereas projectile trauma may permit reconstruction of wound morphology and trajectory, thereby contributing to medicolegal interpretation [11, 12]. This exposure profile is consistent with current definitions of free-roaming cats [13].

From an anatomopathological and forensic standpoint, systematic postmortem examination is essential for documenting traumatic lesions, determining the immediate cause of death, identifying concurrent disease, and supporting inference regarding the mechanism of injury. This is particularly important in cats because external lesions may underestimate the severity of internal damage, especially in cases of blunt force trauma, diaphragmatic rupture, thoracic hemorrhage, or visceral displacement. Moreover, the morphologic classification of injuries into blunt force trauma, sharp-force trauma, and projectile trauma provides a useful framework for correlating lesion patterns with probable injurious agents and for distinguishing accidental from potentially non-accidental trauma when relevant [14–16].

Although feline trauma has been addressed in clinical series and registry-based investigations, studies integrating necropsy findings with gross and microscopic pathologic interpretation remain comparatively limited, particularly in free-roaming cats. In addition, the contribution of concurrent pre-existing disease to traumatic death has received little attention, despite the possibility that debilitating conditions may reduce mobility, impair escape behavior, or increase vulnerability to fatal injury. Therefore, the present study aimed to characterize the gross and histopathologic findings of fatal traumatic injuries in free-roaming cats and to correlate lesion patterns with the predominant traumatic mechanisms observed in a retrospective series of 40 necropsy cases, interpreted within an anatomopathological and forensic framework.

## Materials and methods

### Study design and case selection

A retrospective study was conducted based on archived necropsy records of domestic cats examined at the Veterinary Pathology Laboratory (LPV), Universidade Federal da Paraíba (UFPB), over a 10-year period, from September 2014 through September 2024. All feline necropsy records from this period were reviewed, and cases were screened for evidence of fatal traumatic injury.

Cases were included only when trauma could be established as the primary cause of death based on the integration of gross postmortem findings, histopathologic evaluation, and available clinical and circumstantial information. Only cats that were dead on arrival and submitted directly to LPV/UFPB for necropsy were included. Accordingly, the study was restricted to immediately fatal traumatic events, and no hospitalized cases, treated cases, postsurgical deaths, or animals that underwent clinical stabilization attempts before death were included.

Cases were excluded when the available information was insufficient to establish trauma, rather than the specific precipitating mechanism, as the primary cause of death, or when traumatic lesions were present but considered unrelated to the fatal outcome. This included cases in which trauma was interpreted as incidental to another lethal condition, represented a non-fatal accompanying lesion, or could not be confidently linked to death as the principal fatal event. Iatrogenic and postsurgical lesions were not included. Environmental emergencies such as burns, electrocution, and drowning were not classified as traumatic injuries for the purposes of this study.

### Operational definitions

For the purposes of this study, a fatal traumatic injury was defined as an acute external physical injury that directly resulted in death and was considered the principal lethal event at necropsy.

Immediate death was defined as death occurring at the scene or within a short interval after trauma, before hospital admission, clinical stabilization, or therapeutic intervention, with direct submission of the body for necropsy.

Non-immediate death was defined as death occurring after survival long enough to allow hospitalization, monitoring, surgery, or treatment; such cases were excluded from the study.

Polytrauma was defined as simultaneous traumatic injury involving two or more body regions and/or organ systems.

A confirmed traumatic mechanism was assigned when the precipitating event leading to the fatal trauma was documented in the available history, such as road traffic accident, bite injury, or firearm trauma. A presumed traumatic mechanism was assigned when trauma had already been established as the primary cause of death, and such cases were therefore retained in the study, but the specific precipitating event was not directly documented and could only be inferred with reasonable confidence from lesion morphology, lesion distribution, photographic documentation, radiographic findings when available, and circumstantial information.

An indeterminate traumatic mechanism was assigned when trauma was confirmed as the cause of death, but the specific external event could not be identified with confidence.

A concurrent disease was defined as any non-traumatic lesion or disease process identified at necropsy or histopathology in addition to the traumatic lesions. A pre-existing disease potentially contributing to vulnerability was defined as a non-traumatic condition that could plausibly impair mobility, escape behavior, vision, sensorium, or general physical condition, thereby increasing susceptibility to fatal trauma.

### Clinical and circumstantial data

All available information was retrieved from the diagnostic archive, including original submission forms, raw case records, final necropsy reports, photographic documentation, archived histologic slides, and radiographic examinations when available. Clinical and circumstantial data reviewed included the reported traumatic event, the condition in which the animal was found, the specific traumatic mechanism when documented or presumed from circumstantial information, and any additional remarks recorded at submission.

Because some animals were found dead or were submitted without prior veterinary evaluation, the amount of available historical information varied among cases. However, only cases in which trauma could be confidently established as the primary cause of death were included, even when the specific precipitating event could not be directly documented.

### Study population

All cats included in the study were classified as free-roaming, including semi-domiciled cats with unrestricted outdoor access, stray or rescued cats recovered from the street, and cats with unknown previous ownership history but clear evidence of unrestricted exposure to the outdoor environment, consistent with current feline welfare and population-management definitions of free-roaming cats [10, 13].

Animals were classified according to sex, breed, and age. Age was recorded from the submission records and, whenever possible, converted to months for statistical analysis. For analytical purposes, cats were grouped by age as kittens (0–6 months), juveniles (> 6 months to 2 years), adults (> 2 to 10 years), and geriatric cats (> 10 years) [10].

### Necropsy examination and tissue sampling

Complete necropsies were performed in all cases according to the routine procedures of the LPV/UFPB. All animals underwent systematic external and internal postmortem examination.

Representative tissue samples were routinely collected from all major organs and from all traumatic lesions identified at necropsy. Depending on the distribution of lesions in each case, these included skin and subcutis from representative wounds, skeletal muscle, thoracic wall and ribs when affected, lungs, trachea and bronchi when relevant, diaphragm, heart and great vessels, liver, spleen, kidneys, and segments of the gastrointestinal tract when perforation or hemorrhage was present. Brain and spinal cord were evaluated when head trauma, neurologic injury, or lesions suggestive of central nervous system involvement were identified. Any grossly abnormal tissue suggestive of concurrent disease was also sampled.

### Histopathology

Tissue samples were fixed in 10% neutral buffered formalin, routinely processed, embedded in paraffin wax, sectioned at 4 μm, and stained with hematoxylin and eosin (H&E) for histopathologic examination.

Histopathologic evaluation was used to characterize traumatic lesions and to identify concurrent or pre-existing non-traumatic diseases. When present, microscopic assessment included hemorrhage, edema, atelectasis, pulmonary contusion, skeletal muscle necrosis and hemorrhage, subcutaneous tissue disruption, visceral laceration or perforation, central nervous system hemorrhage or traumatic injury, and any pre-existing lesion considered relevant to the interpretation of the case.

### Trauma classification and lesion assessment

Cases were classified according to the predominant mechanism of injury and the morphologic pattern of the lesions into blunt force trauma, sharp-force trauma, or projectile trauma, based on established veterinary forensic pathology terminology [14–16].

Blunt force trauma comprised lesions attributable to impact, compression, crushing, or other non-sharp external force, typically producing contusions, internal hemorrhage, fractures, and multisystem injury [14]. Sharp-force trauma comprised penetrating injuries produced by the mechanical application of a sharp structure to the body [15]. In the present series, bite-associated cases were included in this category because the predominant wound pattern consisted of puncture or penetrating injuries, although associated tearing, crushing, or other blunt soft tissue damage could also be present in some cases [15]. Projectile trauma comprised injuries caused by ballistic projectiles, including wound tracts associated with tissue penetration and variable secondary blunt tissue effects [16]. Fracture was recorded as gross evidence of skeletal discontinuity at any anatomic site. Because of the retrospective nature of the study, fracture configuration (e.g., simple or comminuted) and detailed topographic subclassification were not analyzed separately. For each case, lesion assessment was based on external wound morphology, internal lesion distribution, and review of available photographic and radiographic documentation. Lesions were recorded according to both topographic distribution and organ-system involvement. Topographic distribution included head/neck, thorax, abdomen, limbs, and multiple regions/polytrauma. Organ-system categories included integumentary, musculoskeletal, thoracic, cardiovascular, abdominal/visceral, and nervous system lesions.

The immediate cause of death was determined by integrating gross findings, histopathologic findings, and available circumstantial information. Major mechanisms of death considered in this assessment included internal hemorrhage/exsanguination, hemorrhagic or hypovolemic shock, traumatic brain injury, respiratory failure secondary to thoracic trauma, and combined fatal polytrauma.

### Concurrent disease assessment

Concurrent non-traumatic lesions were systematically recorded in all cases. When present, these lesions were classified as either incidental or potentially predisposing. A concurrent non-traumatic lesion was defined as any non-traumatic lesion or disease process identified at necropsy or histopathology that was not directly associated with the cause of death. A lesion was considered potentially predisposing when it could reasonably have reduced the animal’s ability to avoid trauma or survive the traumatic event.

### Data collection

When available, radiographs from the diagnostic archive were reviewed and integrated into case interpretation. Imaging findings were used primarily to document fractures, retained projectiles, lesion distribution, diaphragmatic displacement, and internal traumatic damage not fully appreciable on gross inspection alone. Photographic documentation was also reviewed in all available cases to confirm wound morphology, body distribution of lesions, and the relationship between external and internal traumatic findings.

### Necropsy-based traumatic lesion burden index

To provide a standardized descriptive summary of traumatic lesion burden across cases, an exploratory necropsy-based traumatic lesion burden index (EGTN) was developed a priori for this study. The index was designed to summarize the anatomic extent and morphologic complexity of traumatic lesions documented at necropsy using variables consistently available in the case records. It was not intended to estimate lethality or prognosis, but rather to support comparative description of lesion burden across traumatic mechanisms.

Anatomic extent was scored by assigning 1 point for each affected body region (head/neck, thorax, abdomen, and limbs). Critical organ-system involvement was scored by assigning 1 point for each affected major system (thoracic, cardiovascular, abdominal/visceral, and nervous system). The presence of pre-specified major traumatic lesions was scored dichotomously, with 1 point assigned for each lesion category present: hemorrhage, hemothorax, hemoperitoneum, hemopericardium, pulmonary hemorrhage, pulmonary atelectasis/collapse, diaphragmatic rupture, visceral herniation into the thoracic cavity, fracture, and visceral perforation. The total index ranged from 0 to 18 points. As an exploratory descriptive index, the EGTN was used to summarize the overall extent and morphologic complexity of traumatic injury across cases and to support descriptive comparison of lesion burden among traumatic mechanisms.

### Statistical analysis

Data were initially summarized descriptively using absolute and relative frequencies for categorical variables. Continuous variables were summarized using measures of central tendency and dispersion appropriate to their distribution, including mean, median, range, and interquartile range when applicable. Statistical analyses were performed in Python version 3.13.5 using pandas version 2.2.3, SciPy version 1.17.0, and NumPy version 2.3.5.

Descriptive and exploratory inferential comparisons were performed according to trauma category, age group, age in months, EGTN, lesion distribution, and selected gross pathologic findings. Because of the limited sample size, the unbalanced distribution among trauma categories, and the non-Gaussian distribution of the EGTN, inferential analyses were based primarily on nonparametric and exact methods. Associations between categorical variables were assessed using Fisher’s exact test. Correlation between age in months and EGTN was assessed using Spearman’s rank correlation coefficient. Comparisons of EGTN between two groups were performed using the Mann–Whitney U test, whereas comparisons among more than two groups were performed using the Kruskal–Wallis test.

Because only one case was classified as projectile trauma, this category was excluded from inferential comparisons and analyzed descriptively only. In addition, among road-traffic trauma cases in which the type of vehicle could be identified, exploratory subgroup comparisons between car-related and motorcycle-related trauma were performed for EGTN and selected lesion-pattern variables. A significance level of 5% was adopted, and all inferential results were interpreted as exploratory. No adjustment for multiple comparisons was applied because all inferential analyses were exploratory and hypothesis-generating.

## Results

### Study population

During the 10-year study period, 289 feline necropsies were performed at the LPV/UFPB, of which 40 cases (13.84%) were classified as immediately fatal traumatic injuries and included in the study. All cats were classified as free-roaming. Histopathologic examination was available in all cases.

Of the 40 cats, 22 were male and 18 were female. Thirty-eight were mixed-breed and 2 were Siamese (Table 1). With respect to age group, 11 cats were classified as kittens, 10 as juveniles, 17 as adults, 1 as geriatric, and 1 could not be assigned to a defined age category (Table 2).

**Table 1:** Sex and breed of 40 free-roaming cats with immediately fatal traumatic injuries

Characteristic	*n* (%)*	Characteristic	*n* (%)
Sex		Breed	
Male	22 (55.0)	Mixed-breed	38 (95.0)
Female	18 (45.0)	Siamese	2 (5.0)

* Values are presented as number (%) and were calculated from the total study population of 40 cats. Percentages refer to the frequency of each category within the series

**Table 2:** Age group of 40 free-roaming cats with immediately fatal traumatic injuries

Characteristic	*n* (%)
Age group	
Kittens (0–6 months)	11 (27.5)
Juveniles (> 6 months–2 years)	10 (25.0)
Adults (> 2–10 years)	17 (42.5)
Geriatric (> 10 years)	1 (2.5)
Unclassified^#^	1 (2.5)

*#* One cat could not be assigned to a defined age category because age information was insufficient in the archived record

### Trauma categories and traumatic mechanisms

According to the predominant lesion mechanism, 32/40 cases were classified as blunt force trauma, 7/40 as sharp-force trauma, and 1/40 as projectile trauma (Table 3). In all included cases, trauma was confirmed as the primary cause of death; however, the certainty of the specific precipitating mechanism varied according to the available historical and circumstantial information. Mechanism certainty was classified as confirmed in 23 cases, presumed in 8, and indeterminate in 9. Road traffic accident was the most frequent recorded or presumed specific traumatic mechanism (29/40), followed by bite injury (6/40), one confirmed furniture-related crushing trauma case (1/40), and projectile trauma caused by a ballistic projectile (1/40). A small number of blunt force trauma cases could not be linked with certainty to a single specific traumatic event and remained classified as nonspecific blunt force trauma or indeterminate trauma patterns (Table 4). Radiographic examinations, when available, were used as complementary documentation of selected lesions, particularly fractures, diaphragmatic displacement, and retained projectiles, and supported the interpretation of lesion distribution in specific cases.

**Table 3:** Trauma categories and mechanism certainty in 40 free-roaming cats with immediately fatal traumatic injuries

Variable	Category	*n* (%)
Trauma category	Blunt force trauma	32 (80.0)
	Sharp-force trauma	7 (17.5)
	Projectile trauma	1 (2.5)
Mechanism certainty	Confirmed	23 (57.5)
	Presumed*	8 (20.0)
	Indeterminate^**†**^	9 (22.5)

*A traumatic mechanism was classified as “presumed” when it could be inferred with reasonable confidence from lesion pattern and circumstantial information

^**†”**^Indeterminate” indicates that a specific precipitating event could not be assigned with confidence

**Table 4:** Specific traumatic mechanisms in 40 free-roaming cats with immediately fatal traumatic injuries

Specific traumatic mechanism	*n* (%)
Road traffic accident	29 (72.5)
Bite injury	6 (15.0)
Crushing trauma	1 (2.5)
Projectile trauma (ballistic)	1 (2.5)
Nonspecific blunt force trauma	1 (2.5)
Indeterminate trauma	1 (2.5)
Sharp-force trauma by cutting/sharp object	1 (2.5)

### Topographic distribution and major gross findings

Traumatic lesions most frequently involved the thorax (31/40), followed by the abdomen (15/40), head/neck (9/40), and limbs (7/40), whereas multiple body regions were affected in 25/40 cases (Table 5). In terms of organ-system involvement, thoracic lesions predominated (31/40), followed by musculoskeletal (15/40), abdominal/visceral (15/40), nervous system (10/40), integumentary (9/40), and cardiovascular lesions (5/40) (Table 5).

**Table 5:** Topographic distribution and organ-system involvement of traumatic lesions in 40 free-roaming cats with immediately fatal traumatic injuries

Topographic distribution*	*n* (%)	Organ-system involvement	*n* (%)
Head/neck affected	9 (22.5)	Integumentary lesions	9 (22.5)
Thorax affected	31 (77.5)	Musculoskeletal lesions	15 (37.5)
Abdomen affected	15 (37.5)	Thoracic lesions	31 (77.5)
Limbs affected	7 (17.5)	Cardiovascular lesions	5 (12.5)
Multiple regions^**†**^	25 (62.5)	Abdominal/visceral lesions	15 (37.5)
		Nervous system lesions	10 (25.0)

*Topographic regions and organ-system categories were recorded independently for each case; therefore, individual animals could contribute to more than one category, and percentages do not sum to 100%

^**†**^“Multiple regions” denotes simultaneous involvement of more than one topographic region and corresponds to the spatial distribution of polytrauma

**Fig. 1:**
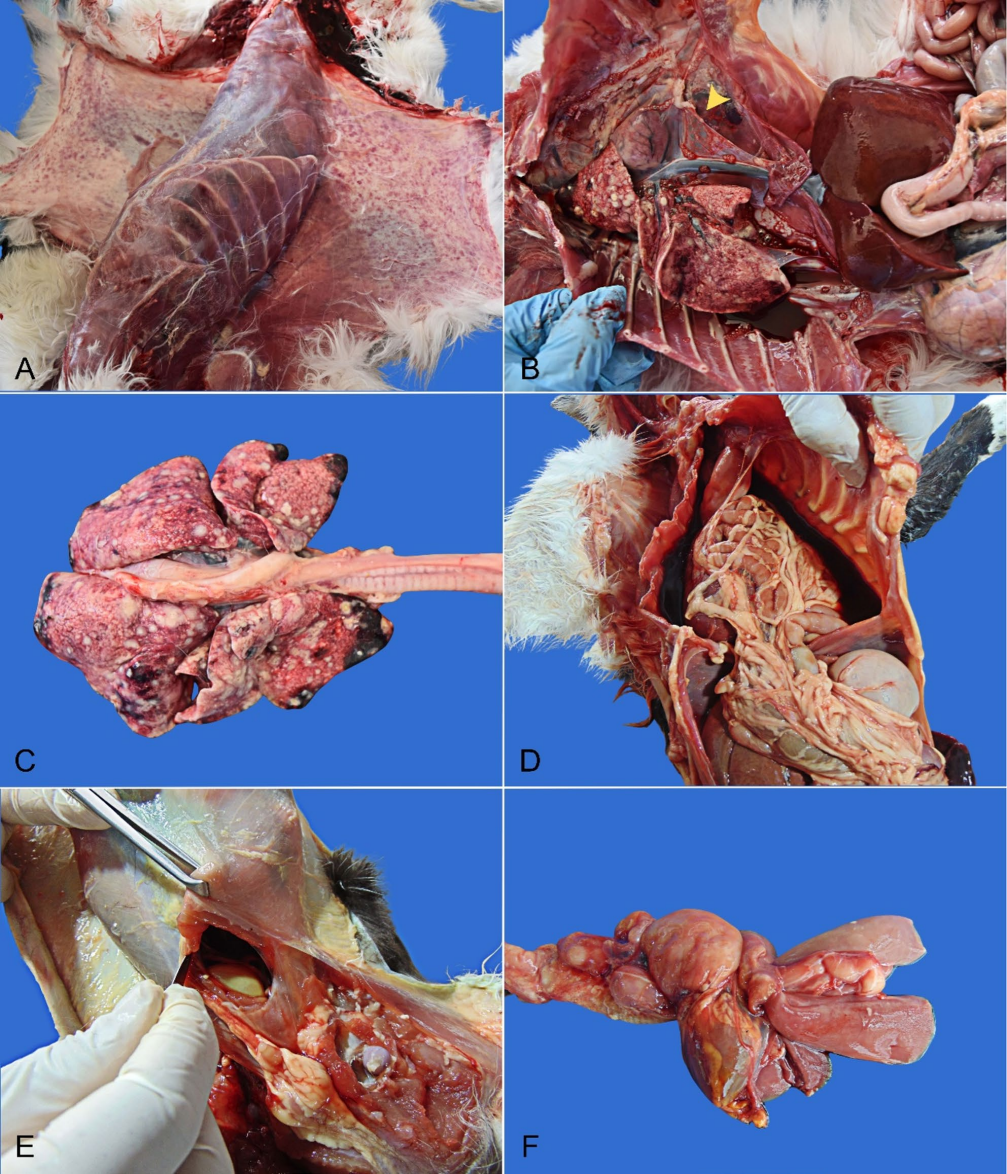
Representative traumatic lesions in free-roaming cats. **A** Multiple punctate to coalescing subcutaneous hemorrhagic foci in the thoracoabdominal region of a 1-year-old male cat with blunt force trauma consistent with roadkill. **B** Traumatic diaphragmatic rupture (arrowhead) in the same cat, with associated pulmonary hemorrhage and multiple white pulmonary nodules. **C** Lungs from the same animal showing multifocal hemorrhage, diffuse edema, and numerous nodular white masses, histologically confirmed as pulmonary adenocarcinoma. **D** Traumatic diaphragmatic hernia in an adult female cat, with intestinal loops, liver, and omentum displaced into the thoracic cavity, associated with hemothorax. **E** Muscular laceration and abdominal wall defect in the same cat, associated with visceral herniation. **F** Marked pulmonary collapse in the same animal

**Fig. 2:**
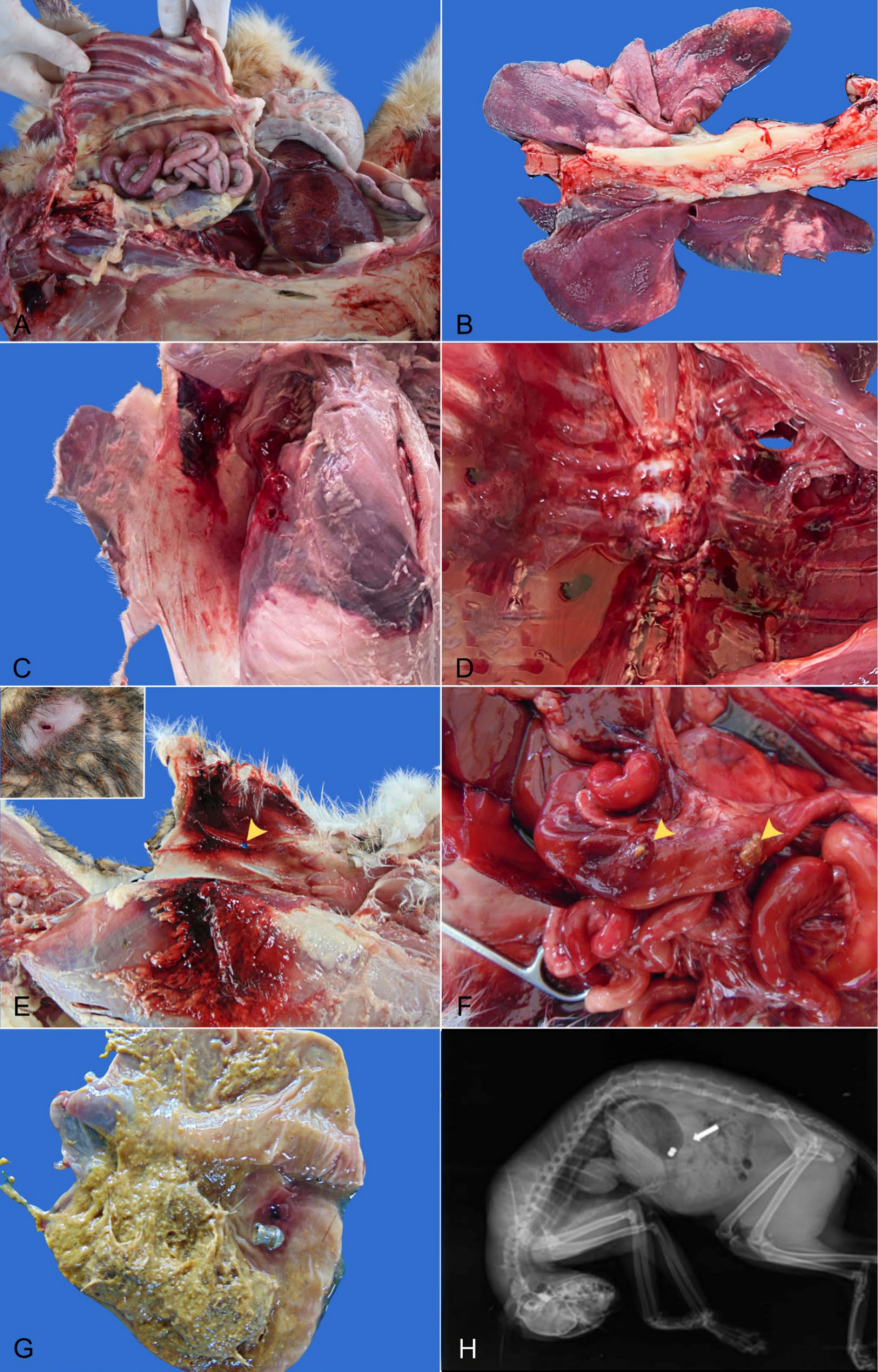
Representative thoracic, thoracoabdominal, bite-associated, and ballistic traumatic lesions in free-roaming cats. **A** Adult male mixed-breed free-roaming cat with traumatic diaphragmatic hernia, showing herniation of the duodenum, jejunum, half of the spleen, and the proximal portion of the pancreas into the thoracic cavity. **B** Lungs from the cat shown in panel **A**, showing marked pulmonary collapse and diffuse dark-red discoloration of the pleural surface. **C** Three-month-old female Siamese free-roaming cat with sharp-force trauma caused by bite injury to the left thoracic region. **D** Thoracic cavity from the cat shown in panel **C**, with sharp-force traumatic lesions involving the thoracic wall and extending to the lung and liver. **E** Three-year-old female mixed-breed free-roaming cat with projectile trauma caused by a ballistic projectile in the abdominal region, showing marked subcutaneous hemorrhage in the ventral abdomen. **F** Intestinal segment from the cat shown in panel E, with projectile perforation of the colon; one perforation is present in the proximal portion, and a second perforation is visible approximately 4 cm caudal to the first (arrowheads). **G** Retained ballistic projectile compatible with a round airgun pellet lodged in the cardia region. **H** Radiographic image of the cat shown in panels **E**–**G**, demonstrating a radiopaque structure (arrow) consistent with a retained ballistic projectile fragment

The most frequent major gross findings in the overall series were extrapulmonary hemorrhage (28/40), pulmonary hemorrhage (20/40), atelectasis or pulmonary collapse (16/40), fracture (16/40), hemothorax (13/40), pulmonary edema (12/40), hemoperitoneum (12/40), visceral herniation into the thoracic cavity (12/40), muscle injury (12/40), diaphragmatic rupture (11/40), deep puncture wounds (9/40), skin laceration (7/40), visceral perforation (5/40), and hemopericardium (4/40) (Table 6). Because road traffic accident was the predominant recorded or presumed traumatic mechanism in the series, these overall frequencies were driven largely by blunt force trauma cases, which showed the broadest and most complex lesion patterns. In particular, road-traffic-associated blunt force trauma was characterized by frequent thoracic involvement with multiregional extension and accounted for most instances of pulmonary hemorrhage, pulmonary collapse, fracture, hemothorax, hemoperitoneum, diaphragmatic rupture, and visceral herniation into the thoracic cavity, supporting a predominant pattern of severe thoracoabdominal polytrauma in immediately fatal cases. Bite-associated sharp-force trauma, by contrast, showed a more localized morphologic profile, characterized mainly by deep puncture wounds, skin lacerations, and muscle injury, most often involving the cervicothoracic or thoracic regions. The single projectile trauma case showed a focal ballistic pattern with abdominal penetration, visceral perforation, internal hemorrhage, and retained projectile material. Because of the limited number of bite-associated and projectile cases, these patterns were interpreted descriptively rather than inferentially. Representative images of the different traumatic lesions observed in the cats included in this study are compiled in Figs. 1 and 2.

**Table 6:** Major gross traumatic findings by trauma category in 40 free-roaming cats with immediately fatal traumatic injuries

Gross finding	Blunt force trauma (*n* = 32), n (%)	Sharp-force trauma (*n* = 7), n (%)	Projectile trauma (*n* = 1), n (%)	Total (*n* = 40), n (%)	*P* value*
Extrapulmonary hemorrhage	25 (78.1)	2 (28.6)	1 (100.0)	28 (70.0)	0.020
Hemothorax	13 (40.6)	0 (0.0)	0 (0.0)	13 (32.5)	0.073
Hemoperitoneum	11 (34.4)	0 (0.0)	1 (100.0)	12 (30.0)	0.152
Hemopericardium	4 (12.5)	0 (0.0)	0 (0.0)	4 (10.0)	0.562
Pulmonary hemorrhage	19 (59.4)	1 (14.3)	0 (0.0)	20 (50.0)	0.044
Pulmonary edema	11 (34.4)	1 (14.3)	0 (0.0)	12 (30.0)	0.641
Atelectasis/pulmonary collapse	16 (50.0)	0 (0.0)	0 (0.0)	16 (40.0)	0.029
Diaphragmatic rupture	11 (34.4)	0 (0.0)	0 (0.0)	11 (27.5)	0.151
Visceral herniation into thoracic cavity	12 (37.5)	0 (0.0)	0 (0.0)	12 (30.0)	0.077
Skin laceration	5 (15.6)	2 (28.6)	0 (0.0)	7 (17.5)	0.586
Deep puncture wounds	4 (12.5)	4 (57.1)	1 (100.0)	9 (22.5)	0.022
Muscle injury	9 (28.1)	3 (42.9)	0 (0.0)	12 (30.0)	0.652
Fracture	16 (50.0)	0 (0.0)	0 (0.0)	16 (40.0)	0.029
Visceral perforation	2 (6.2)	2 (28.6)	1 (100.0)	5 (12.5)	0.120

*P values refer to exploratory comparisons between blunt force trauma and sharp-force trauma only, using Fisher’s exact test. The single projectile trauma case was excluded from inferential comparisons and is shown descriptively only

### Necropsy-based traumatic lesion burden index

The exploratory necropsy-based traumatic lesion burden index (EGTN) ranged from 0 to 16 points in the 40 cases analyzed (mean, 6.50; median, 7.0; interquartile range, 4.0–9.0). Because this index was designed to summarize lesion burden rather than lethality itself, it was interpreted only as a descriptive estimate of anatomic extent and lesion complexity. Blunt force trauma cases showed broader anatomic extent, reflected by higher EGTN values (mean, 7.47; median, 7.0; range, 2–16), whereas sharp-force trauma cases showed lower overall EGTN values (mean, 2.29; median, 3.0; range, 0–4) (Table 7). The single projectile trauma case had an EGTN of 5. No significant association was detected between age and lesion burden index, whether age was analyzed as a continuous variable (Spearman’s rho = 0.127, *P* = 0.435) or by age group (Kruskal–Wallis, *P* = 0.750). Blunt force trauma cases also had significantly higher EGTN values than sharp-force trauma cases (Mann–Whitney U, *P* = 0.00031), supporting the greater spatial extent and morphologic complexity of blunt force injury in this series. Among road-traffic trauma cases in which the type of vehicle could be identified, car-related trauma more often showed multiregional injury (12/13 vs. 2/7; Fisher’s exact test, *P* = 0.0072) and hemoperitoneum (8/13 vs. 0/7; *P* = 0.0147) than motorcycle-related trauma. Car-related trauma also showed higher EGTN values than motorcycle-related trauma (mean, 9.38 vs. 5.43; Mann–Whitney U, *P* = 0.0119), whereas abdominal involvement was numerically more frequent but did not reach statistical significance (6/13 vs. 0/7; *P* = 0.0515).

**Table 7:** Exploratory necropsy-based traumatic lesion burden index (EGTN) according to trauma category

Trauma category*	Mean	Median	Range
Blunt force trauma (*n* = 32)	7.47	7.0	2–16
Sharp-force trauma (*n* = 7)	2.29	3.0	0–4

*The single projectile trauma case had an EGTN of 5 and was excluded from group comparisons; this case is presented descriptively in the text only

### Concurrent non-traumatic lesions and potentially predisposing conditions

Concurrent non-traumatic lesions were recorded in 5/40 cases (Table 8). These included pulmonary adenocarcinoma, cutaneous squamous cell carcinoma (SCC), multicentric lymphoma, cholangiocarcinoma associated with cholangitis, and platynosomosis due to *Platynosomum fastosum*. In addition, 9/40 cases were flagged during case review as having potentially predisposing pre-existing conditions or other recorded factors considered relevant to increased vulnerability. Most of these cases involved chronic debilitating or neoplastic conditions that may have impaired general condition, mobility, or the ability to avoid traumatic injury.

**Table 8:** Concurrent non-traumatic lesions recorded in free-roaming cats with immediately fatal traumatic injuries

Case ID	Trauma category	Concurrent diagnosis recorded	Potentially predisposing**	Recorded rationale
CAT14	Blunt force trauma	Mucopurulent rhinitis; rhinotracheitis	Yes	Systemic debility/cachexia/rhinotracheitis
CAT16	Blunt force trauma	Pulmonary adenocarcinoma; paraneoplastic syndrome	Yes	Systemic debility/functional limitation
CAT29	Blunt force trauma	Auricular SCC* with extension to bone and meninges	Yes	Systemic debility/functional limitation/possible neurologic involvement
CAT30	Blunt force trauma	Multicentric lymphoma	Yes	Systemic debility/functional limitation
CAT31	Blunt force trauma	Nasal SCC; cholangiocarcinoma; cholangitis; platynosomosis; strongyloidiasis	Yes	Systemic debility/functional limitation

*Squamous cell carcinoma

**“Potentially predisposing” refers to pre-existing non-traumatic conditions judged capable of impairing general condition, mobility, or escape behavior, thereby increasing vulnerability to traumatic death

## Discussion

The principal finding of this study is that immediately fatal trauma in free-roaming cats was characterized by distinct lesion patterns according to traumatic mechanism, with road-traffic-associated blunt force trauma representing the dominant and most informative pattern in the series. Free-roaming cats are exposed to multiple external hazards in urban environments [17], particularly under conditions of unrestricted exposure to vehicular traffic [2], which likely contributes to the high frequency of severe blunt force trauma observed in this species [1–4, 8, 12]. This pattern is consistent with previous clinical and epidemiologic studies identifying trauma as an important cause of death in cats and vehicular blunt force trauma as the predominant mechanism in feline trauma populations [2–4, 8]. However, the present study extends those observations by focusing specifically on immediately fatal cases submitted directly for necropsy, thereby providing a detailed anatomopathologic perspective on the lesion patterns associated with lethal trauma in free-roaming cats. In this context, the predominance of blunt force trauma in our series reinforces the central role of high-energy impact events in feline traumatic death and highlights the value of systematic postmortem examination for documenting the full extent of internal injury, which may be substantially greater than suggested by external lesions alone [5].

Road-traffic-associated blunt force trauma represented the largest and most informative subset of the present series and was characterized predominantly by thoracic involvement, frequent multiregional extension, pulmonary hemorrhage, pulmonary collapse, fractures, hemothorax, hemoperitoneum, diaphragmatic rupture, and visceral herniation into the thoracic cavity. This distribution is biologically plausible and consistent with the mechanics of high-energy impact injury, in which kinetic energy is transmitted across the thorax and cranial abdomen, often producing severe internal damage even when external lesions appear comparatively limited [1–6, 8, 12]. Thus, the findings more importantly show that fatal road-traffic trauma in free-roaming cats tends to present as a pattern of severe thoracoabdominal polytrauma with complex multisystem involvement, which helps explain its strong representation among immediately fatal cases [2–5, 8].

In the present fatal necropsy series, the predominance of thoracic involvement, together with the high frequency of multiregional injury, diaphragmatic rupture, and visceral herniation, indicates that severe thoracoabdominal trauma was strongly represented among immediately fatal cases. This interpretation is biologically plausible in free-roaming cats, which are regularly exposed to hazardous outdoor behaviours such as road crossing and encounters with other animals, thereby increasing the likelihood of high-energy impact events [17, 18]. Once blunt force trauma occurs, rapid transmission of kinetic energy to the thorax and cranial abdomen may produce pulmonary hemorrhage, collapse, and complex thoracoabdominal injury, while abrupt changes in the pressure gradient between the pleural and peritoneal cavities can lead to diaphragmatic rupture and migration of abdominal organs into the thoracic cavity [5, 19, 20]. Previous studies in cats have consistently linked traumatic diaphragmatic hernia to vehicular trauma and have identified the liver, stomach, and small intestine among the organs most commonly displaced, which is broadly consistent with the lesions observed in our cases [20–22]. In this context, the high proportion of multiregional injury in our study reinforces the interpretation that many of these animals were subjected to high-energy blunt-force impact rather than focal trauma, which helps explain why thoracic lesions frequently coexisted with abdominal, musculoskeletal, and, in some cases, neurologic injury [1, 4, 5].

Sharp-force trauma, although less frequent than blunt force trauma in the present series, showed a distinct and forensically informative lesion pattern, characterized mainly by deep puncture wounds, skin lacerations, and muscular injury. This profile is consistent with bite-associated trauma in cats, in which external wounds may appear relatively limited in size but can be associated with severe underlying tissue disruption, vascular damage, pneumothorax, and fatal internal injury [9, 23]. In our cases, the predominance of deep puncture wounds in the sharp-force group, together with the mainly cervicothoracic and thoracic distribution of lesions, supports the interpretation that this category reflects a more localized but still potentially lethal traumatic mechanism. From a forensic perspective, this wound pattern is particularly relevant because paired punctures, opposing wound distribution, and associated soft tissue damage may help distinguish bite trauma from other forms of penetrating or blunt injury and may support reconstruction of the mechanism of injury during postmortem investigation [5, 9]. Accordingly, despite its more localized distribution, sharp-force trauma showed a consistent and highly informative morphologic signature in fatal feline trauma cases.

Concurrent non-traumatic lesions and potentially predisposing conditions also deserve attention in the interpretation of fatal feline trauma. In our series, a subset of cats had concurrent diseases, including neoplastic and other debilitating conditions, which may have impaired general condition, mobility, or responsiveness and thereby increased vulnerability to fatal injury. This issue may be particularly relevant in younger free-roaming animals, since kittens and juveniles together represented more than half of the study population, and previous feline studies have shown that younger cats are disproportionately represented among trauma- and road traffic accident-related deaths [2, 24]. Although our retrospective design does not allow direct determination of whether abandonment or lack of prior supervision contributed to individual traumatic events, this possibility should not be overlooked, especially in free-roaming or unowned kittens. In such animals, physical immaturity, limited environmental experience, and unrestricted exposure to outdoor hazards may plausibly increase susceptibility to severe trauma, while the broader welfare literature also recognizes free-roaming and unowned kittens as a particularly vulnerable subgroup requiring specific management and protective strategies [17, 25].

Projectile trauma was represented by a single case and therefore cannot support broader inference regarding recurrent lesion distribution. Even so, this case illustrated a recognizable focal ballistic pattern, with abdominal penetration, visceral perforation, internal hemorrhage, retained projectile material, and radiographic confirmation of the foreign body. Such lesions remain important from a forensic standpoint because wound morphology, projectile recovery, and imaging findings may contribute directly to reconstruction of trajectory and mechanism of injury in individual cases [5, 11].

The exploratory necropsy-based traumatic lesion burden index (EGTN) provided a useful framework for summarizing the anatomic extent and morphologic complexity of traumatic injury across cases, particularly in this necropsy-based series dominated by blunt force polytrauma. In the present study, higher EGTN values were observed mainly in blunt force trauma and in car-related road traffic accidents, consistent with the broader distribution of lesions and greater frequency of multiregional injury in those groups. Because the revised EGTN was constructed as a simple additive descriptive index, based on affected body regions, critical organ-system involvement, and the presence of pre-specified major traumatic lesions, it should be interpreted as a complementary tool for summarizing overall lesion burden rather than as a hierarchy of biological severity among individual lesions. In this context, the EGTN helped summarize lesion burden across traumatic mechanisms while remaining secondary to direct lesion-pattern analysis [4–6].

The present findings also underscore the forensic relevance of systematic necropsy in fatal feline trauma. Careful postmortem examination allows not only determination of the immediate cause of death, but also reconstruction of the probable mechanism of injury through recognition of lesion distribution and wound morphology. This is particularly important in cats because external lesions may underestimate the severity of internal trauma, especially in blunt force trauma, while patterned lesions such as paired puncture wounds, localized soft tissue disruption, and ballistic tracts may provide strong clues regarding bite trauma, projectile injury, or other specific mechanisms [5, 9, 11, 23]. In this context, standardized documentation of gross findings, photographic evidence, radiographic support when available, and correlation with histopathology are highly relevant to forensic interpretation. Beyond diagnostic value, these approaches also have implications for animal welfare and legal investigation, since some traumatic deaths may raise concern for neglect, deliberate injury, or other forms of non-accidental trauma, making the pathologist’s role central to both case clarification and future protective action [5, 11, 26].

Although the present study provides a detailed necropsy-based characterization of immediately fatal trauma in free-roaming cats, some aspects may be further refined in future investigations. Because this was a retrospective series restricted to fatal cases submitted directly for necropsy, the findings are particularly informative for understanding lesion patterns associated with lethal trauma; however, larger multicenter studies and comparative datasets including non-fatal cases may help broaden the clinical and epidemiologic interpretation of these patterns [3, 4, 12]. Likewise, more complete integration of scene information, clinical records when available, and ancillary diagnostic findings may further improve mechanism classification in cases in which the traumatic event cannot be directly confirmed. Future studies may also expand the comparative evaluation of lesion patterns across different traumatic mechanisms and further examine descriptive approaches for summarizing lesion burden in broader and more heterogeneous trauma populations. Taken together, such efforts would contribute to a more comprehensive understanding of feline trauma, further expand the applicability of the present findings, and reinforce the role of veterinary pathology in trauma characterization, animal welfare assessment, medicolegal investigation, and advancement of the existing literature [5, 9, 26, 27].

## Conclusion

In conclusion, immediately fatal trauma in free-roaming cats was dominated by blunt force trauma, most often associated with road traffic accidents, and characterized by extensive thoracic involvement, frequent multiregional distribution, and severe internal damage. Road-traffic-associated cases showed a predominant pattern of thoracoabdominal polytrauma, whereas bite-associated sharp-force trauma and projectile trauma showed more localized lesion profiles. The systematic necropsy-based approach adopted in this study, supported by histopathology and complementary imaging when available, allowed detailed characterization of lethal lesion patterns, recognition of concurrent non-traumatic conditions, and more consistent interpretation of probable traumatic mechanisms. Taken together, these findings reinforce the value of veterinary pathology not only in the diagnosis of fatal feline trauma, but also in the broader contexts of forensic interpretation, animal welfare assessment, and prevention-oriented understanding of trauma in free-roaming cat populations.

## Data Availability

Data Availability Statement​The data supporting the findings of this study are included within the article. Additional information may be made available from the corresponding author upon reasonable request.

## Abbreviations

AAFP: American Association of Feline Practitioners
EGTN: Exploratory necropsy-based traumatic lesion burden index
H&E: Hematoxylin and eosin
LPV: Veterinary Pathology Laboratory
SCC: Squamous cell carcinoma
UFPB: Universidade Federal da Paraíba
